# Integrating single cell analysis and machine learning methods reveals stem cell-related gene S100A10 as an important target for prediction of liver cancer diagnosis and immunotherapy

**DOI:** 10.3389/fimmu.2024.1534723

**Published:** 2025-01-07

**Authors:** Shenjun Huang, Tingting Tu

**Affiliations:** ^1^ Department of Oncology, Nantong Tumur Hospital (Affiliated Tumur Hospital of Nantong University), Nantong, China; ^2^ Department of Radiation Oncology, Lianyungang Second People’s Hospital (Lianyungang Tumur Hospital), Lianyungang, China

**Keywords:** cancer stem cell, hepatocellular carcinoma, single cell analysis, machine learning, S100A10

## Abstract

**Background:**

Hepatocellular carcinoma (LIHC) poses a significant health challenge worldwide, primarily due to late-stage diagnosis and the limited effectiveness of current therapies. Cancer stem cells are known to play a role in tumor development, metastasis, and resistance to treatment. A thorough understanding of genes associated with stem cells is crucial for improving the diagnostic precision of LIHC and for the advancement of effective immunotherapy approaches.

**Method:**

This research combines single-cell RNA sequencing with machine learning techniques to identify vital stem cell-associated genes that could act as prognostic biomarkers and therapeutic targets for LIHC. We analyzed various datasets, applying negative matrix factorization alongside machine learning algorithms to reveal gene expression patterns and construct diagnostic models. The XGBoost algorithm was specifically utilized to identify key regulatory genes related to stem cells in LIHC, and the expression levels and prognostic significance of these genes were validated experimentally.

**Results:**

Our single-cell analysis identified 16 differential prognostic genes associated with liver cancer stem cells. Cluster analysis and diagnostic models constructed using various machine learning techniques confirmed the significance of these 16 genes in the diagnosis and immunotherapy of LIHC. Notably, the XGBoost algorithm identified S100A10 as the stem cell-related gene most relevant to the prognosis of LIHC patients. Experimental validation further supports S100A10 as a potential prognostic marker for this cancer type. Additionally, S100A10 shows a positive correlation with the stem cell marker POU5F1.

**Conclusion:**

The results of this study highlight S100A10 as an essential predictor for liver cancer diagnosis and treatment response, particularly regarding immunotherapy. This research offers valuable insights into the molecular mechanisms underlying LIHC and suggests S100A10 as a promising target for enhancing treatment outcomes in liver cancer patients.

## 1 Introduction

Liver hepatocellular carcinoma (LIHC) is the most prevalent type of primary liver cancer, with approximately 800,000 new cases diagnosed each year (1, 2). It is the sixth most common cancer globally and the third leading cause of cancer-related deaths (3). The unfavorable prognosis of LIHC is largely due to late-stage diagnosis (4). Despite the availability of various treatment options—including surgical resection, chemotherapy, radiofrequency or microwave ablation, molecular targeted therapies, and immunotherapy—clinical outcomes for advanced LIHC have not significantly improved (5). Immunotherapy has shown promise as a treatment modality; however, its effectiveness is often limited by the scarcity of viable targets, impacting only a subset of patients (6). Identifying immune-related prognostic biomarkers is essential for recognizing patient subgroups that may benefit from immunotherapy, underscoring the necessity for further research into additional biomarkers.

The combination of single-cell analysis and machine learning algorithms has emerged as a valuable approach for identifying key genes that regulate tumor progression, particularly regarding diagnostics and immunotherapy (7, 8). This innovative methodology facilitates a detailed examination of the complex tumor microenvironment (TME) and aids in uncovering significant molecular factors that contribute to the heterogeneity and treatment resistance observed in LIHC. Single-cell RNA sequencing (scRNA-seq) offers a comprehensive perspective on the cellular makeup of tumors, facilitating the discovery of unique cell types that play roles in tumor onset, development, and spread. Through the application of sophisticated computational methods like machine learning, scientists can better interpret intricate biological data, which aids in pinpointing potential crucial genes. This progress is essential for tailoring cancer treatments and developing innovative immunotherapy approaches (9–11). The investigation of liver cancer, especially in LIHC, has increasingly centered on cancer stem cells (CSCs) because of their significant involvement in tumor development, metastasis, and the resistance to conventional treatments (12). The heterogeneity of liver CSCs, characterized by various surface markers, complicates the disease and presents challenges for diagnosis and treatment. A comprehensive understanding of CSC-related genes is essential for improving diagnostic precision and developing effective immunotherapy strategies (13). CSCs are believed to enhance tumor self-renewal and proliferation, a phenomenon that is especially evident in LIHC, where a small fraction of cells exhibiting stem cell-like properties can differentiate and play a role in tumor diversity. This diversity is associated with differing treatment responses, such as to immunotherapy, underscoring the importance of targeted strategies that consider the unique traits of CSCs. The shift from an epithelial to a mesenchymal phenotype (EMT), which is associated with increased malignancy and invasiveness in tumors, particularly LIHC, is governed by signaling pathways like transforming growth factor beta and Wnt/β-catenin. These pathways are essential for maintaining the stem-like features of liver cancer cells (14, 15). Monitoring circulating CSCs can provide insights into LIHC recurrence and may serve as potential biomarkers for immunotherapy response, as these cells often exhibit unique immunogenic profiles that can be targeted therapeutically. Recent studies have identified specific CSC-related genes that may act as prognostic markers for LIHC. For instance, the stemness index (mRNAsi) has been used to categorize LIHC patients into subtypes based on their stemness signatures, which correlate with the status of the tumor immune microenvironment (TIME) and sensitivity to neoadjuvant therapies. Such classification could inform clinical strategies for immunotherapy, leading to more personalized treatment plans that take into account the unique stemness characteristics of individual tumors (16). The immune microenvironment in liver cancer is significantly affected by CSCs. Interactions between CSCs and immune cells can result in immune evasion, presenting a major challenge to effective cancer treatment. CSCs can secrete factors that modulate immune responses, fostering an environment conducive to tumor growth and survival. This underscores the importance of targeting CSCs in immunotherapy, as strategies aimed at enhancing immune responses against these cells may improve treatment outcomes (17). Furthermore, identifying immune-related gene signatures associated with CSCs can assist in predicting the efficacy of immunotherapy in LIHC patients. By analyzing gene expression profiles, researchers have developed predictive models for patient survival and response to immune checkpoint inhibitors, leveraging the understanding of how CSC-related genes interact with the immune system to establish a framework for personalized therapeutic approaches (18).

In conclusion, CSC-related genes play a critical role in the diagnosis and treatment of liver cancer, particularly in the context of immunotherapy. Their involvement in tumor heterogeneity, immune evasion, and therapeutic resistance necessitates a deeper understanding of their functions and interactions within the TME. By integrating CSC-related markers into diagnostic and treatment strategies, clinicians can enhance the precision of liver cancer management and ultimately improve patient outcomes. Ongoing research into the molecular mechanisms governing CSCs and their relationship with the immune system is expected to lead to more effective immunotherapeutic strategies for LIHC. This study aims to investigate the key genes regulating liver cancer stem cells using various methods, including single-cell analysis and machine learning. Additionally, we seek to analyze the diagnostic and predictive capabilities of stem cell-related genes for liver cancer patients by constructing a diagnostic model. Ultimately, we identified the critical role of S100A10 through the XGBoost algorithm and assessed the expression and prognostic significance of S100A10 using immunofluorescence staining.

## 2 Materials and methods

### 2.1 Datasets and patient samples

This research analyzed three LIHC specimens (GSM3064824, GSM3064820, and GSM3064823) sourced from the GSE112271 dataset at the resolution of single cells. Furthermore, we merged RNA sequencing data alongside clinical information derived from the TCGA-LIHC dataset. In order to create and validate the diagnostic model, several datasets were employed, comprising TCGA-LIHC, GSE112790, and GSE102451. The clinical prognostic data of 240 primary liver cancer samples from Japan in the IGCG database were also included in this study.

### 2.2 Negative matrix factorization cluster and differential expression analysis

The NMF algorithm was utilized to derive coefficients of biological significance from the gene expression matrix, arranging genes and samples to emphasize the structural properties of the data and aid in classification (19). Differential expression analysis for clusters A and B was carried out utilizing the ‘Limma’ R package, with the parameters established at |logFC| > 0.5 and an adjusted p-value lower than 0.05. Following this, the ‘NMF’ R package was employed to group all samples according to the differentially expressed genes (DEGs) found in the subclusters, with the goal of uncovering potential molecular subtypes. The ‘brunet’ algorithm was performed for 100 iterations for each specified value, varying from 2 to 10 clusters. The ideal number of clusters was identified by assessing cophenetic correlation, dispersion, and silhouette width (20). Furthermore, the Limma package in R (version 3.40.2) was used to analyze mRNA differential expression between malignant and adjacent non-malignant tissues in the TCGA-PRAD dataset.

### 2.3 Immune infiltration analysis

To validate the reliability of the immune score results, we utilized the immunedeconv R package (21). An extensive assessment of every algorithm was conducted, highlighting their distinct benefits. The XCELL approach was chosen due to its ability to evaluate a wider variety of immune cell categories (22).

### 2.4 Constructing the diagnostic model

The training phase employed the TCGA-LIHC dataset, while validation was conducted using the GSE112790 and GSE102451 datasets. Model combinations were evaluated based on their area under the curve (AUC) values, identifying the optimal model as the one with the highest average AUC. Receiver operating characteristic (ROC) curve analysis was performed using the pROC package [1.18.0], with results visualized via ggplot2 [3.3.6].

### 2.5 Differential and gene function analysis

The Limma package (version 3.40.2) in R was utilized to explore the differential expression of mRNA within the TCGA-LIHC dataset. We set thresholds for identifying differentially expressed mRNAs at “P < 0.05 and log2 (fold change) > 1.3 or log2 (fold change) < -1.3.” To further investigate the roles of target genes in carcinogenesis, we employed the ClusterProfiler package to analyze Gene Ontology (GO) functions and to enrich Kyoto Encyclopedia of Genes and Genomes (KEGG) pathways. Relevant pathway-associated genes were compiled and analyzed using the GSVA package in R, and single-sample gene set enrichment analysis (ssGSEA) was conducted with the method parameter set to ‘ssgsea’. Additionally, we evaluated the correlation between gene expression and pathway scores using Spearman correlation analysis.

### 2.6 Expression and prognostic relevance of S100A10 in LIHC tissue microarrays analyzed by immunofluorescence methods

After initially baking the paraffin slices, they were immersed in two xylene baths, each lasting 15 minutes. This was followed by a series of consecutive soaks in absolute ethanol, 95% ethanol, 85% ethanol, 75% ethanol, and finally distilled water, with each solution applied for 5 minutes. The sections were then transferred to a retrieval box containing an alkaline antigen retrieval solution (pH 9.0 EDTA) and heated in a pressure cooker for 2 minutes. After allowing the sections to cool naturally, they were washed three times with PBS (pH 7.4) for 5 minutes each, with gentle stirring. Subsequently, the sections were treated with a 3% hydrogen peroxide solution for 15 minutes at room temperature in the dark to inhibit endogenous peroxidase activity. To ensure uniform tissue coverage, a blocking solution was applied, and the sections were blocked for 30 minutes at room temperature. Following this, diluted S100A10 antibody (11250-1-AP) and POU5F1 antibody (60242-1-Ig) were added and incubated overnight at 4°C. The next day, the sections were washed three times with PBS for 5 minutes each. After gently shaking to remove excess liquid, a poly-HRP secondary antibody corresponding to the primary antibody species was added dropwise and incubated in the dark at room temperature for 10 to 20 minutes. The final score of the staining result was calculated by multiplying the staining intensity by the staining range. The staining range was categorized as 0%-25% for 1 point, 26%-50% for 2 points, 51%-75% for 3 points, and 76%-100% for 4 points, while the staining intensity was classified into low, medium, and strong, corresponding to 1, 2, and 3 points, respectively.

### 2.7 Statistical analysis

The expression levels of S100A10 in both LIHC and normal tissues were assessed using the Wilcoxon rank-sum test. Prognostic analysis was performed using the log-rank test to evaluate survival differences. Additionally, Spearman correlation analysis was conducted to explore the relationship between gene expression and stemness scores. A p-value of less than 0.05 was established as the threshold for statistical significance.

## 3 Result

### 3.1 Identification of cancer stem cell-related genes via single-cell analysis

**Figure 1:**
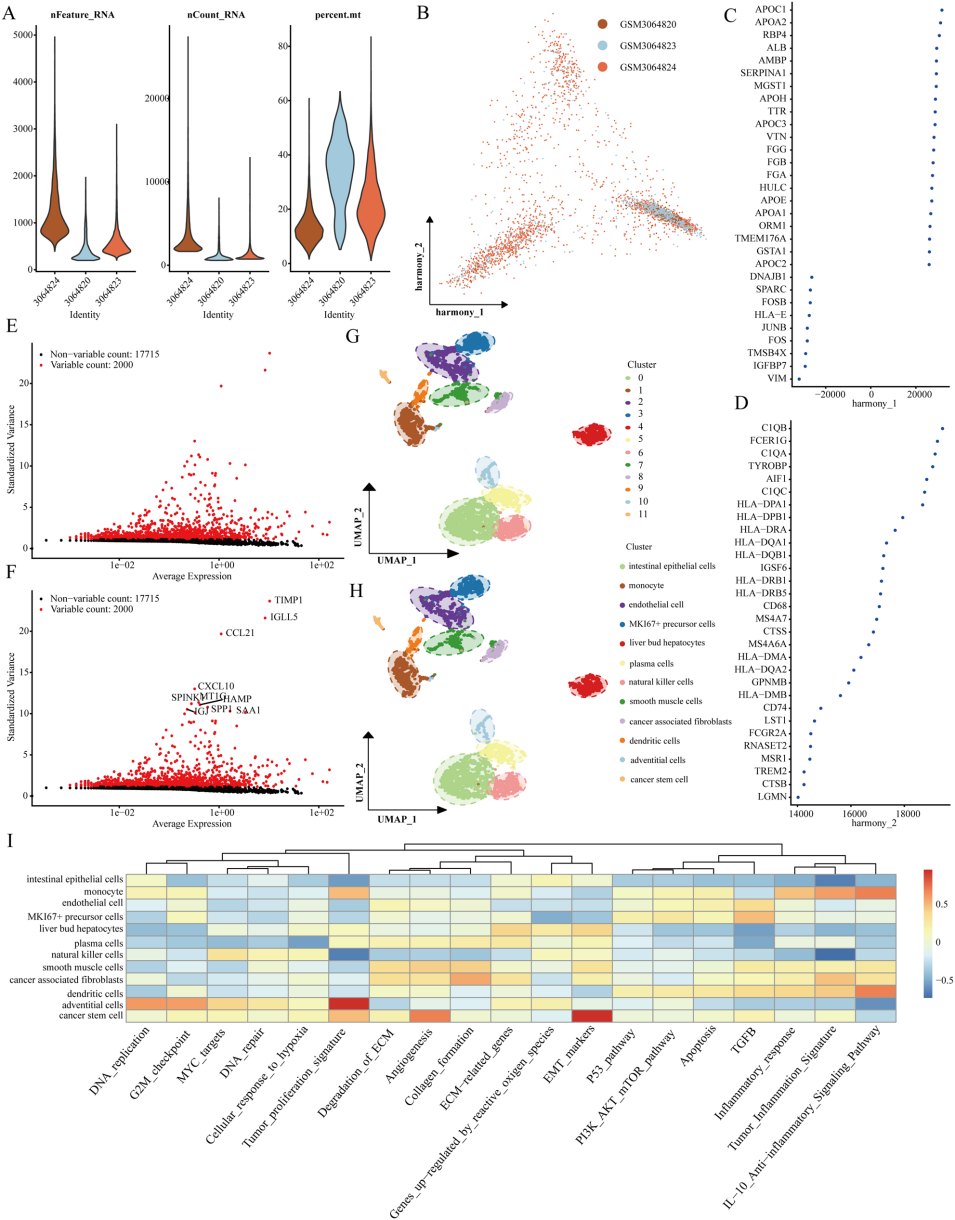
Recognition of genes that signify cancer stem cells. **(A)** Evaluation of the quality of scRNA-seq across different cellular sub-populations. **(B–D)** Visual representations from PCA analysis following the comprehensive removal of batch effects. **(E, F)** Identification of highly variable genes achieved through batch removal post-count. **(G, H)** Categorization of LIHC samples employing the UMAP method. **(I)** Functional assessment of various cellular populations.

Our investigation commenced with three LIHC samples sourced from the single-cell dataset (GSE112271): GSM3064824, GSM3064820, and GSM3064823. Stringent quality control measures were implemented, requiring each cell to contain a minimum of 200 RNA molecules, a maximum of 2500, and less than 10% mitochondrial RNA (**Figure 1A**). Following this, we employed HARMONY technology to pinpoint highly variable genes from the filtered dataset and conducted bulk deletion analysis based on these feature sets (**Figures 1B–D**). The ANOVA test highlighted ten genes with significant differential expression across the cell samples: TIMP1, IGLL5, CCL21, CXCL10, SPINK1, MT1G, HAMP, SPP1, IGJ, and SAA1 (**Figures 1E, F**). The single-cell analysis categorized the samples into 12 distinct cell populations, including natural killer cells, liver bud hepatocytes, smooth muscle cells, plasma cells, MKI67+ precursor cells, endothelial cells, monocytes, intestinal epithelial cells, cancer stem cells, adventitial cells, dendritic cells, and cancer-associated fibroblasts (**Figures 1G, H**). Notably, functional analysis indicated that stem cell populations are linked to processes such as tumor proliferation, angiogenesis, and epithelial-mesenchymal transition (EMT) (**Figure 1I**).

### 3.2 Functional analysis of stem cell-related genes

**Figure 2:**
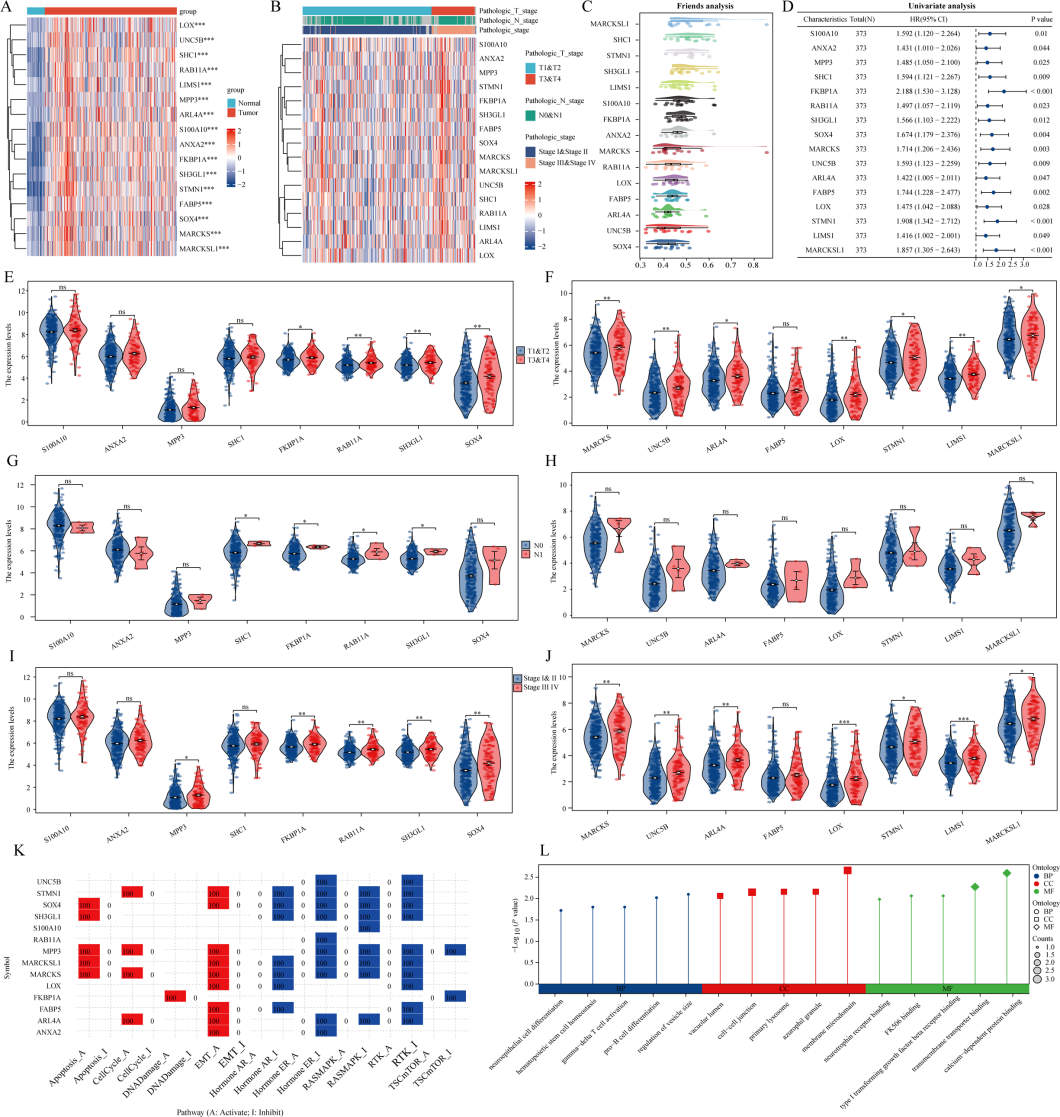
The expression and roles of genes associated with stem cells in the TCGA-LIHC dataset. **(A)** A heatmap illustrates the differential expression of prognostic genes linked to monocytes in both LIHC and normal tissues. **(B)** A separate heatmap showcases the expression levels of genes related to stem cells across various pathological parameters. **(C)** The analysis of friends identifies essential genes among those associated with stem cells. **(D)** An examination of prognostic factors looks at the co-expression of genes related to stem cells. **(E–J)** A violin plot represents the expression patterns of stem cell-related genes across different clinicopathological factors. **(K, L)** An analysis of functionality is performed on these stem cell-associated genes. ns = P > 0.05, *P<0.05, **P<0.01, ***P<0.001

Initially, we examined expression heatmaps of 16 genes in both TCGA-LIHC samples and normal prostate tissue (**Figure 2A**). We further assessed the relationship between these genes and the clinicopathological features of LIHC patients, visualized through a heat map (**Figure 2B**). The Friends analysis aimed to construct a gene interaction network, utilizing network topology to evaluate gene importance, with MARCKSL1 emerging as a central figure (**Figure 2C**). Univariate COX regression analysis in the TCGA-LIHC dataset demonstrated the prognostic relevance of these 16 genes (**Figure 2D**). We found significant expression differences among SOX4, SH3GL1, RAB11A, FKBP1A, ARL4A, UNC5B, MARCKS, MARCKSL1, LIMS1, STMN1, and LOX across various T phases. Moreover, expression levels of SH3GL1, RAB11A, FKBP1A, and SHC1 differed significantly at varying N stages, and MPP3, SOX4, SH3GL1, RAB11A, FKBP1A, ARL4A, UNC5B, MARCKS, MARCKSL1, LIMS1, STMN1, and LOX showed significant variation across different clinical stages (**Figures 2E–J**). Utilizing the Gene Set Cancer Analysis (GSCA) database, we explored the roles of these 16 genes in LIHC, revealing their involvement in EMT, activation of the cell cycle, and inhibition of estrogen receptor and receptor tyrosine kinase (RTK) pathways (**Figure 2K**). Gene Ontology (GO) analysis indicated that these genes were predominantly associated with calcium-dependent protein binding, membrane microdomain, and pro-B cell differentiation (**Figure 2L**).

### 3.3 Clustering analysis

**Figure 3:**
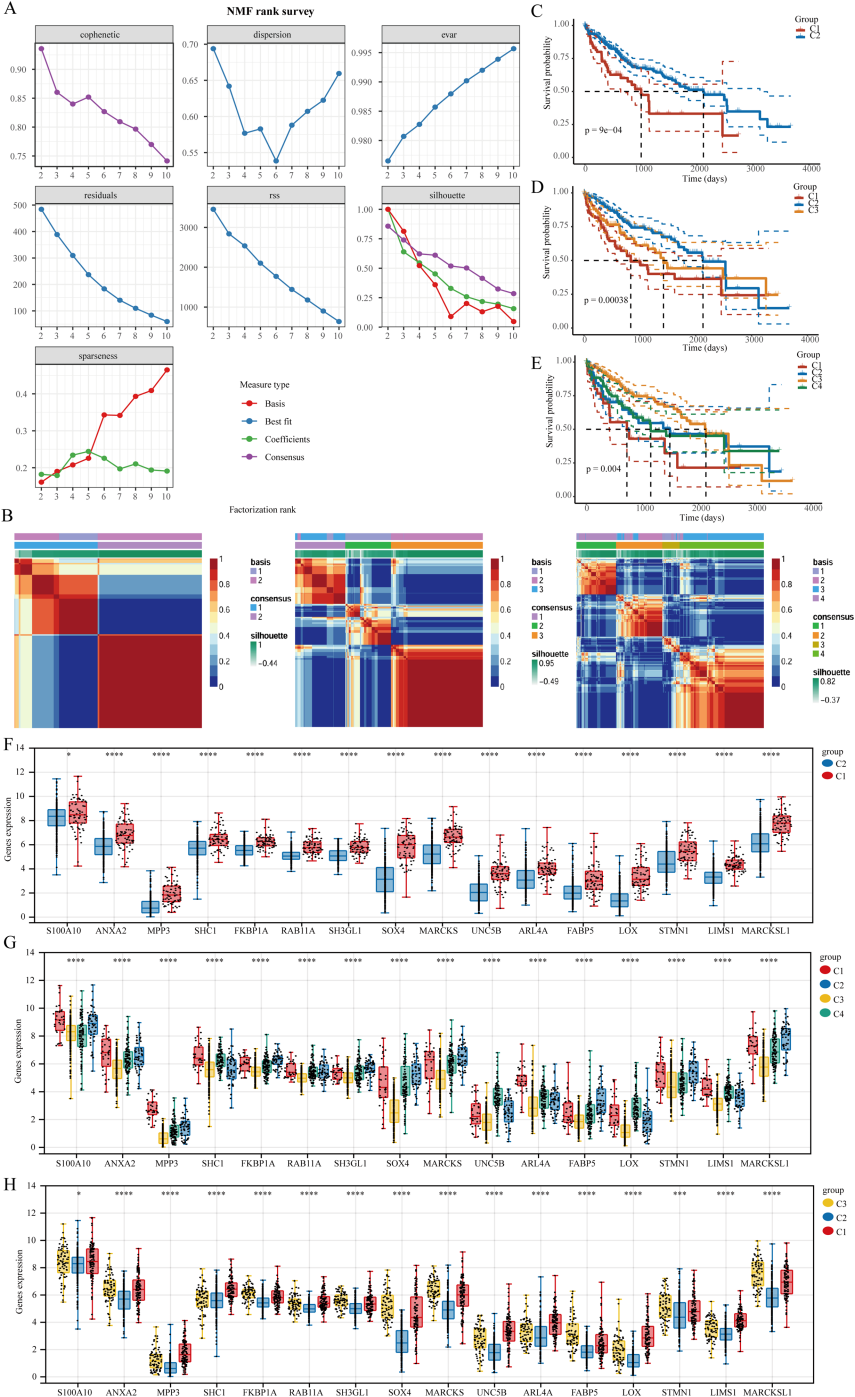
The clustering of LIHC samples utilizing the NMF cluster analysis technique. **(A)** Evaluate cluster stability and performance using various methods. **(B)** Heat map representing NMF clustering. **(C–E)** Differences in prognosis among the clusters. **(F–H)** Variations in the expression of stem cell-related genes across distinct clusters. *P<0.05, ***P<0.001, ****P<0.0001.

To cluster TCGA-LIHC samples, we applied the NMF clustering technique. Co-expression curves were analyzed to determine the optimal classification method for TCGA-LIHC subgroups. The optimal grouping was indicated by the point where the co-expression index exhibited a pronounced decrease. Our results suggested that dividing the samples into two groups was most suitable. Heatmaps illustrating sample divisions into two, three, and four groups are presented (**Figures 3A, B**). We further examined prognostic differences among the groups categorized into two, three, and four clusters. Regardless of the division, patients in cluster 1 consistently demonstrated the poorest prognosis, with p-value analysis confirming that a two-group division was optimal (**Figures 3C–E**). Differential expression of the 16 CSC-related genes across the clusters was also illustrated (**Figures 3F–H**).

### 3.4 Functional analysis of cancer stem cell-related genes

**Figure 4:**
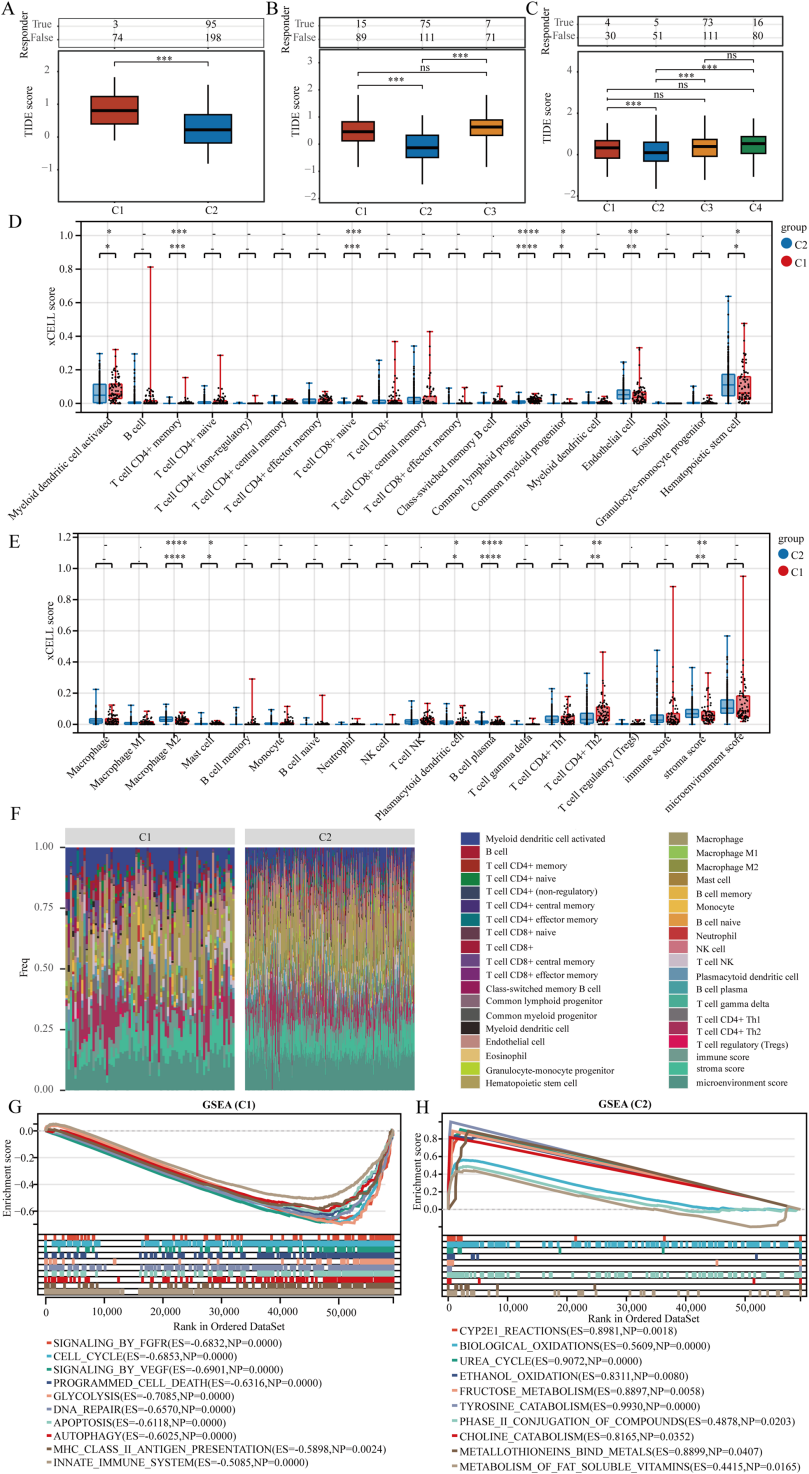
Genes indicative of stem cells correlate with immune cell infiltration in LIHC. **(A–C)** The TIDE algorithm was utilized to evaluate patient responses to immunotherapy across clusters. **(D, E)** Assessment of immune cell infiltration variance in various clusters was conducted using the xCELL algorithm. **(F)** A heat map illustrating immune cell scores. **(G, H)** Analysis of gene enrichment among different clusters. ns = P > 0.05, *P<0.05, **P<0.01, ***P<0.001, ****P<0.0001.

Immune checkpoint blockade (ICB) therapy has transformed cancer treatment. In this study, we utilized the TIDE algorithm, which focuses on tumor immune dysfunction and exclusion, to predict the effectiveness of immune checkpoint inhibitors for each TCGA-LIHC sample (**Figure 4A**). TIDE assesses two mechanisms of tumor immune evasion: damage to tumor-infiltrating cytotoxic T lymphocytes (CTLs) and CTL resistance to immunosuppressive factors. Elevated TIDE scores correlate with diminished ICB effectiveness and reduced survival rates following ICB treatment. Upon clustering TCGA-LIHC samples into two groups, we observed differences in ICB response, with patients in cluster C1 showing poorer responses. In three or four cluster categorizations, patients in cluster C2 exhibited better responses to ICB therapy (**Figures 4B, C**). We also employed xCell to analyze immune cell infiltration levels across the TCGA-LIHC samples in different clusters, revealing significant differences in various immune cell types, including CD4+ memory T cells, naive CD8+ T cells, common lymphoid progenitors, M2 macrophages, and plasma B cells (**Figures 4D–G**).

### 3.5 Integration of machine learning algorithms for diagnostic model development

**Figure 5:**
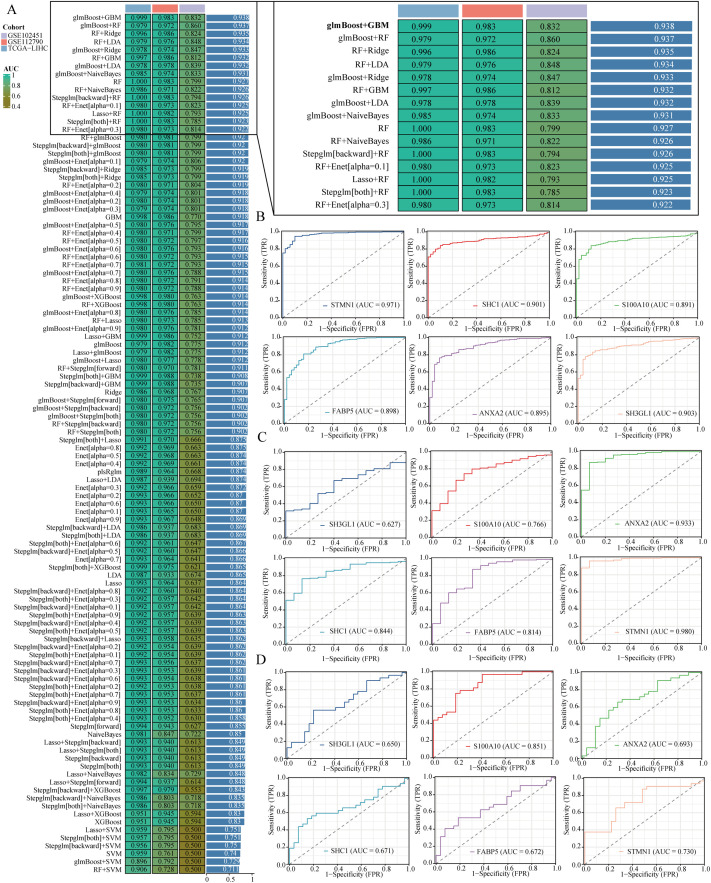
Building a diagnostic model. **(A)** It shows a comparison of area under the curve (AUC) values for diagnostic models created using different combinations of algorithms. **(B–D)** The ROC curves illustrate the effectiveness of various genes in forecasting liver hepatocellular carcinoma (LIHC) within the TCGA-LIHC, GSE112790, and GSE102451 datasets.

Machine learning is increasingly pivotal in biomedicine, particularly in tumor diagnosis and treatment. By analyzing genomic data from patients, machine learning can facilitate personalized treatment approaches. In this study, we developed a diagnostic model centered on liver hepatocellular carcinoma (LIHC) to aid in the early identification of affected individuals. The model was trained using the TCGA-LIHC dataset and validated with two additional datasets: GSE112790 and GSE102451. Among the 113 tested algorithm combinations, the glmBoost+GBM pair demonstrated the highest efficacy for model construction. For reader convenience, we expanded the prediction results of the top 15 algorithm combinations (**Figure 5A**). The area under the curve (AUC) for the TCGA-LIHC training data was 0.999, while the corresponding AUC values for the validation datasets GSE112790 and GSE102451 were 0.983 and 0.832, respectively. The glmBoost+GBM algorithm identified six key genes: STMN1, SHC1, S100A10, FABP5, ANXA2, and SH3GL1. We subsequently presented the individual predictive values of these genes in TCGA-LIHC, GSE112790, and GSE102451 (**Figures 5B–D**).

### 3.6 Role of S100A10 in regulating LIHC stemness

**Figure 6:**
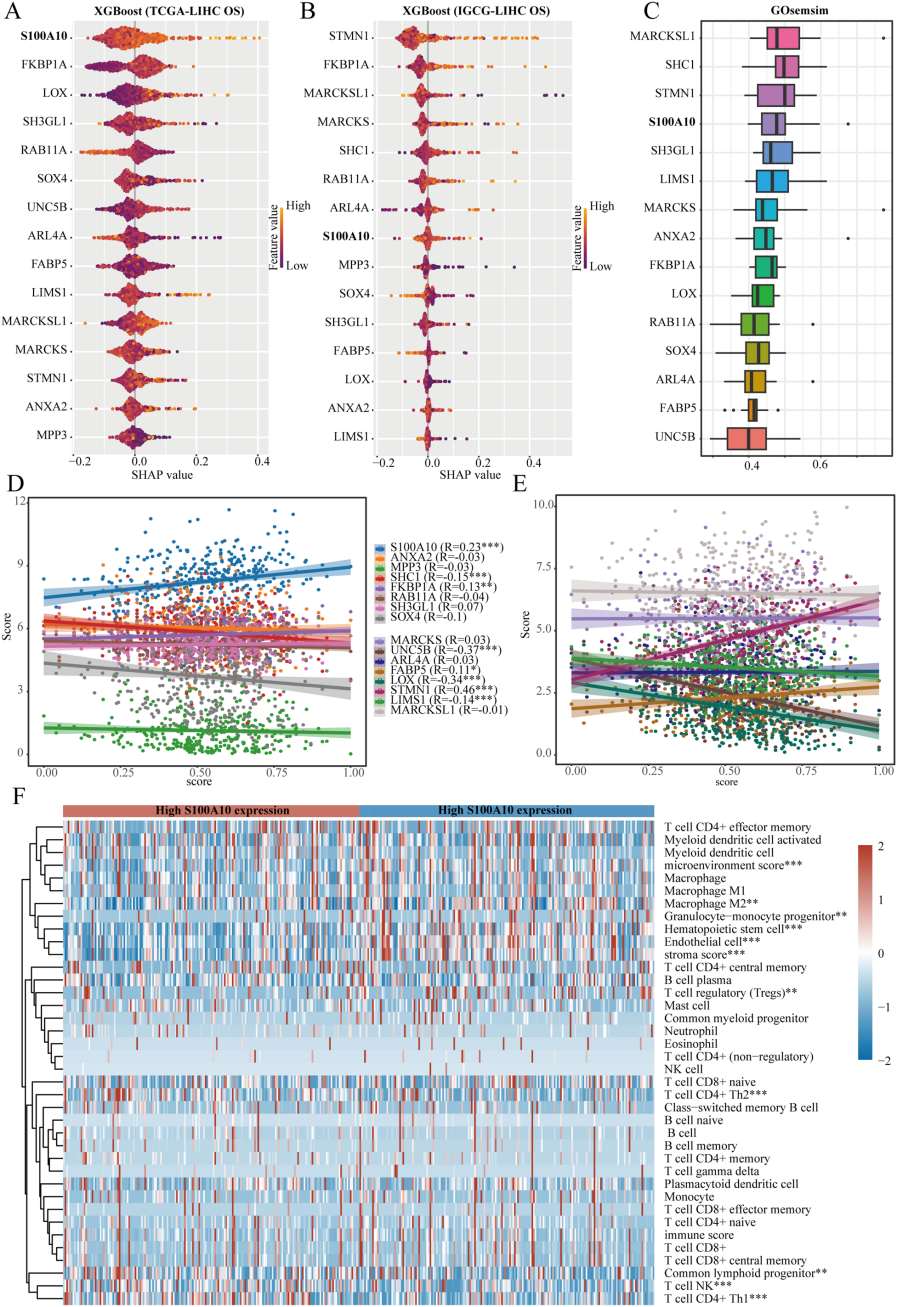
S100A10 as a crucial gene. **(A, B)** The XGBoost algorithm pinpoints the 15 genes that show the strongest association with overall survival (OS) in liver hepatocellular carcinoma (LIHC). **(C)** GOsemSim analysis underscores important genes linked to stem cell traits. **(D, E)** A correlation analysis is performed to evaluate the relationship between a range of genes and the stemness score within the TCGA-LIHC dataset. **(F)** Furthermore, another correlation analysis investigates the connection between S100A10 and the degree of immune cell infiltration in LIHC. **P<0.01, ***P<0.001.

Utilizing the XGBoost algorithm, we analyzed the prognostic significance of stem cell-related genes in relation to clinical data from the TCGA-LIHC and IGCG-LIHC datasets to assess their impact on overall survival. Consistently, S100A10, FKBP1A, RAB11A, SOX4, and ARL4A ranked among the top 10 genes in both datasets (**Figures 6A, B**). GOsemSim employs Gene Ontology annotation data to evaluate similarities between gene sets based on shared functional terms. Among these five genes, S100A10 emerged as the most significant (**Figure 6C**). To quantify tumor stemness in LIHC samples, we utilized a logistic regression-based machine learning algorithm (OCLR) described in a Cell article, which computes the stemness index for various samples. Our findings indicated that S100A10, FKBP1A, FABP5, and STMN1 positively correlated with the stemness score of LIHC samples, underscoring the pivotal role of S100A10 in LIHC stemness characteristics (**Figures 6D, E**). Additionally, we analyzed the correlation between S100A10 expression and immune cell infiltration in the LIHC microenvironment, revealing significant differences in microenvironment scores and levels of M2 macrophages, hematopoietic stem cells, endothelial cells, CD4+ Th2 T cells, and NK T cells between high and low expression groups of S100A10 (**Figure 6F**).

### 3.7 Functional analysis of S100A10 in LIHC

**Figure 7:**
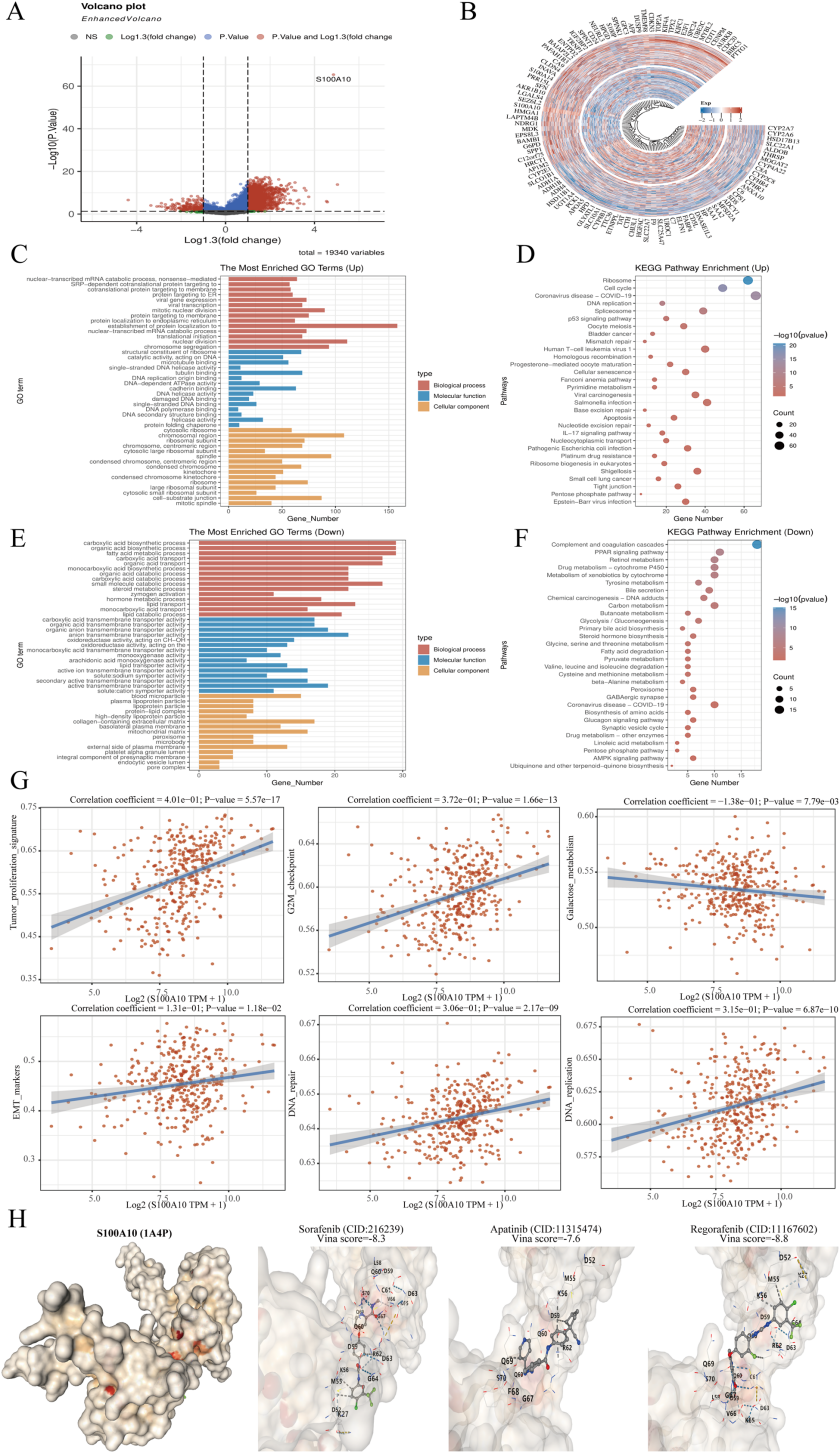
Analysis of S100A10 functionality. **(A)** Volcano plot depicting variance analysis. **(B)** Circular map illustrating differential gene expression. **(C–F)** Examination of S100A10’s functional role in PRAD utilizing KEGG and GO pathways. **(G)** S100A10 function assessment through the ssGSEA algorithm. **(H)** Molecular docking study of S100A10 with frequently used pharmaceuticals in LIHC.

The samples within the TCGA-LIHC dataset were divided according to the median expression of S100A10. Those exhibiting expression levels above this median were designated as the high S100A10 expression group, whereas samples with expression below the median were categorized in the low S100A10 expression group (**Figures 7A, B**). To explore the functional implications of S100A10, GO analysis was conducted on differentially expressed genes. S100A10 showed the strongest associations with tubulin binding and cadherin binding in the molecular function (MF) module among the upregulated genes. Additionally, it was linked to the processes of establishing protein localization to the endoplasmic reticulum and nuclear division within the biological process (BP) module. In terms of the cellular component (CC) module, it was connected to cell−substrate junctions and chromosomal regions. On the other hand, among the downregulated genes, S100A10 showed associations with anion transmembrane transporter activity and active transmembrane transporter activity in the MF category, along with involvement in carboxylic acid biosynthesis and organic acid biosynthetic processes in the BP category, and connections to the collagen−containing extracellular matrix and mitochondrial matrix in the CC category, reflecting a very strong relationship. Utilizing KEGG enrichment analysis is an effective approach for unraveling gene functions and providing valuable genomic insights. Importantly, S100A10 demonstrated significant relationships with ribosome and cell cycle pathways among genes that were highly expressed, whereas it was primarily linked to complement and coagulation cascades, as well as the PPAR signaling pathway among genes that were less expressed (**Figures 7C–F**). Subsequently, the ssGSEA algorithm was utilized to systematically compute the enrichment scores for each TCGA-LIHC sample across different pathways, thus establishing a connection between the samples and their corresponding pathways. By evaluating the relationship between gene expression and pathway scores, our goal was to clarify how each gene relates to its associated pathway. The analysis disclosed that S100A10 expression showed a positive correlation with tumor proliferation, G2M checkpoint activity, epithelial-mesenchymal transition (EMT), DNA replication, and DNA repair processes, whereas a negative correlation was found with galactose metabolism (**Figure 7G**). In addition, we investigated the interaction of S100A10 with therapeutic agents for LIHC, and the findings demonstrated that S100A10 has a substantial binding affinity to these drugs, underscoring its potential as a therapeutic target for liver cancer (**Figure 7H**).

### 3.8 Correlation analysis between S100A10 and stem cell markers

**Figure 8:**
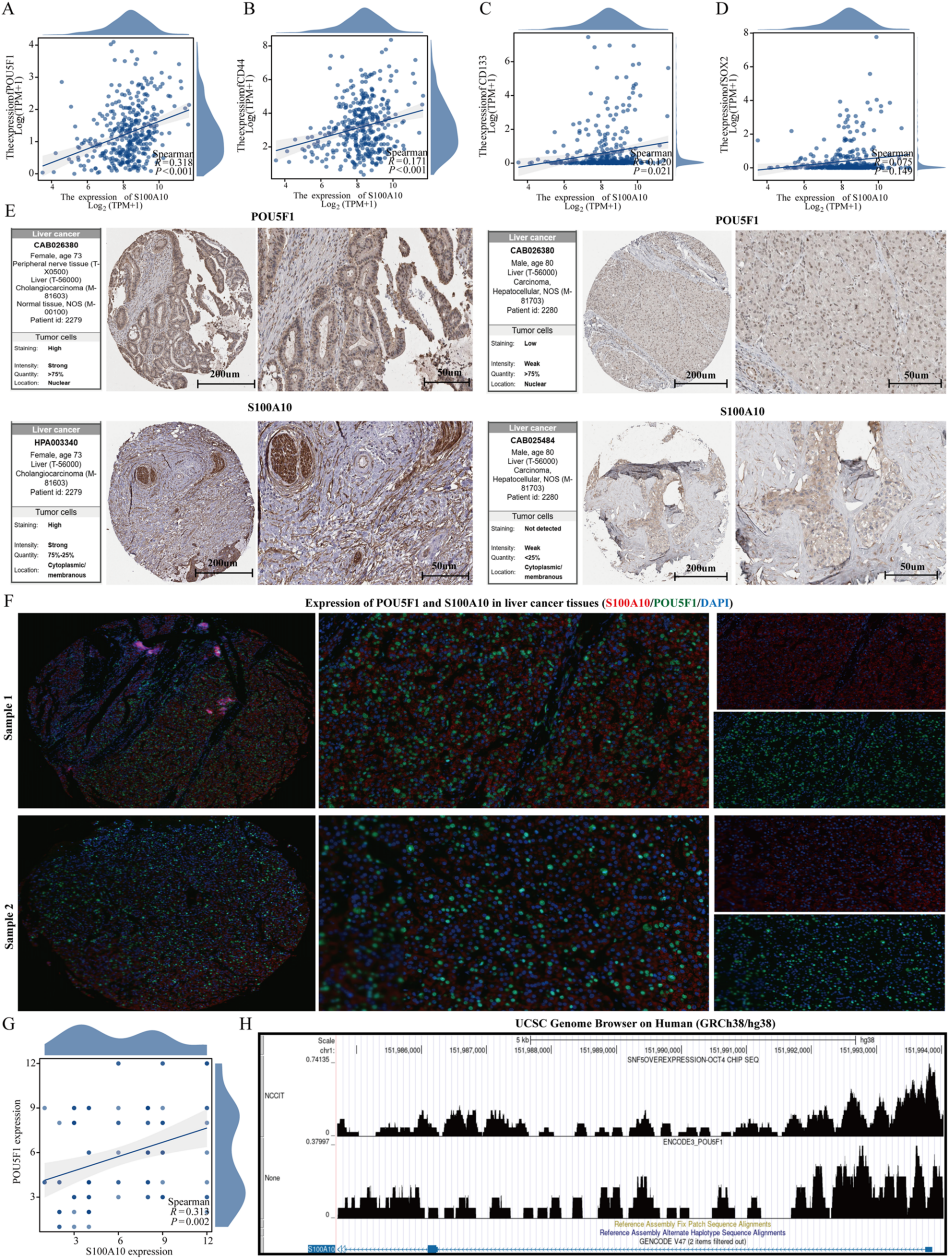
S100A10 is positively correlated with POU5F1. **(A–D)** Correlation analysis between S100A10 and stem cell markers. **(E)** Expression of S100A10 and POU5F1 proteins. **(F, G)** Analysis of correlation between S100A10 and POU5F1 protein expression. **(H)** Analysis of transcriptional regulation of S100A10 and POU5F1.

In the TCGA-LIHC dataset, we analyzed the correlation between stem cell markers (SOX2, CD44, CD133, and POU5F1) and S100A10. Our findings revealed a significant positive correlation between the expression of S100A10 and CD44, CD133, and POU5F1, while no significant correlation was observed with SOX2 (**Figures 8A–D**). Notably, the expression of S100A10 exhibited the strongest correlation with POU5F1, prompting us to further investigate the relationship between these two proteins. Utilizing The Human Protein Atlas database, we discovered a correlation between the expression levels of S100A10 and POU5F1 at the protein level; however, the limited number of cases precluded statistical significance (**Figure 8E**). To enhance our analysis, we collected 185 liver cancer samples (92 from liver cancer patients and 93 from normal liver tissues) from Nantong Cancer Hospital. This analysis confirmed a positive correlation trend between the expression of S100A10 and POU5F1 at the protein level (**Figures 8F, G**). Lastly, given that POU5F1 is a transcription factor, we examined the potential transcriptional regulatory relationship between S100A10 and POU5F1. Our results indicated that POU5F1 was significantly enriched in the promoter region of S100A10 (**Figure 8H**).

### 3.9 S100A10 may serve as a prognostic marker in patients with LIHC

**Figure 9:**
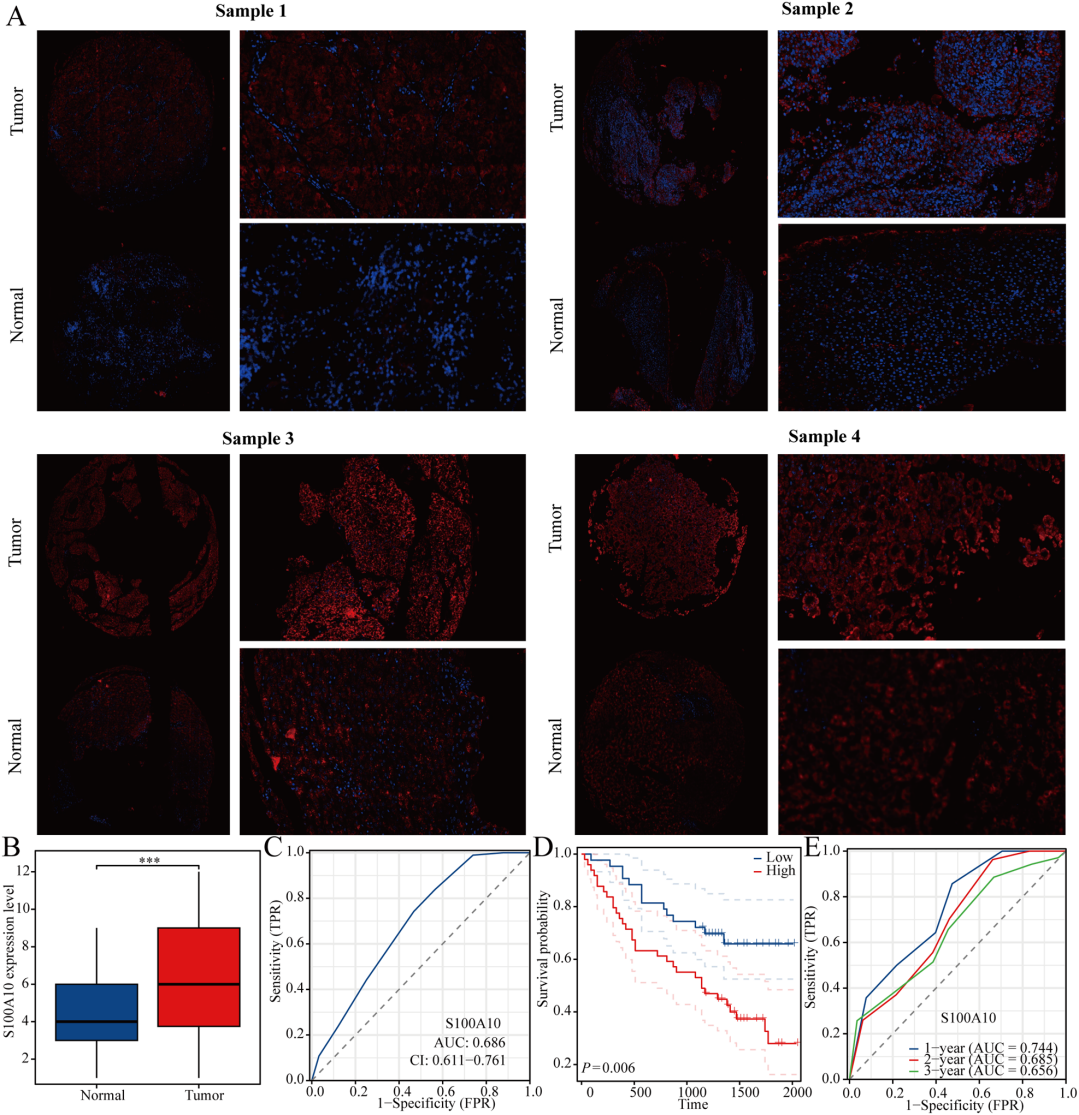
S100A10 is highly expressed in LIHC. **(A, B)** Expression of S100A10 in LIHC. **(C)** ROC curve of S100A10 in predicting liver cancer diagnosis. **(D)** KM curve of S100A10. **(E)** ROC curve of S100A10 in predicting the prognosis of liver cancer. ***P<0.001.

We also analyzed the expression levels of S100A10 in liver cancer samples, revealing that its expression was significantly higher in liver cancer compared to normal liver tissue (**Figures 9A, B**). Additionally, we assessed the predictive value of S100A10 for diagnosing liver cancer using the ROC curve. Our findings indicated that S100A10 possesses a notable predictive value for liver cancer diagnosis (AUC=0.686) (**Figure 9C**). Furthermore, the KM curve analysis demonstrated that patients with elevated S100A10 expression have a poorer prognosis, and S100A10 expression can also serve as a predictor for the 1-year, 2-year, and 3-year prognosis of liver cancer patients (**Figures 9D, E**).

## 4 Discussion

Liver hepatocellular carcinoma (LIHC) poses a serious threat to patient health, characterized by high morbidity and mortality rates (23). Despite recent advancements in treatment modalities, the overall effectiveness of liver cancer therapies remains inadequate. Cancer stem cells (CSCs), a distinct subset of tumor cells, possess self-renewal and differentiation capabilities, which contribute to tumor heterogeneity and resistance to standard treatments (24). These CSCs are critical in the initiation, progression, and recurrence of liver cancer, making them a pivotal focus for developing innovative therapeutic strategies (25). This study aims to analyze the importance of CSC-related genes in the diagnosis, prognosis, and immunotherapy of LIHC, utilizing single-cell technology and machine learning approaches. The outcomes seek to unveil new therapeutic targets and provide a theoretical framework for treating LIHC.

CSCs are defined by their abilities to self-renew and differentiate, which play a vital role in the heterogeneity of tumors and the resistance seen towards conventional treatment methods. It is thought that liver cancer stem cells (LCSCs) arise from either the reversal of mature hepatocytes to a stem-like state or from the impaired differentiation of normal liver stem cells (26). This dual lineage implies that LCSCs can form in various cellular contexts within the liver, making it more challenging to decipher their role in tumor development. They are involved in essential processes associated with liver cancer, including tumor formation, metastasis, and recurrence, primarily because of their inherent abilities to self-renew and differentiate (27). A major challenge presented by LCSCs is their resistance to traditional treatments like chemotherapy and radiotherapy. This resistance can be attributed to various factors, including the expression of drug efflux transporters, which actively remove chemotherapeutic agents from cells, thereby diminishing drug effectiveness. Additionally, LCSCs often exhibit enhanced DNA repair mechanisms and anti-apoptotic traits, allowing them to withstand treatments that effectively eliminate non-stem cancer cells (28). The presence of LCSCs correlates with a poor prognosis in liver cancer patients, with studies indicating that elevated levels of CSC markers are associated with increased tumor aggressiveness and a higher recurrence rate post-treatment. For example, patients with tumors displaying high CD133 expression typically have shorter overall survival compared to those with lower levels (29). This highlights the necessity of targeting LCSCs to improve treatment outcomes and reduce the risk of relapse. LCSCs are implicated in activating several signaling pathways that facilitate tumor growth and metastasis. Notably, the WNT/β-catenin signaling pathway is crucial for sustaining the self-renewal and proliferation of cancer stem cells, and its activation is linked to increased tumorigenicity and invasiveness in liver cancer (30). Targeting these pathways may provide a therapeutic avenue to reduce the stemness of cancer cells and enhance the efficacy of existing treatments. Recent investigations have also highlighted the promise of differentiation induction therapy as a novel strategy for targeting LCSCs. By promoting the differentiation of LCSCs into more mature and less invasive cell types, it is possible to diminish their tumorigenic potential and improve patient outcomes. For instance, inhibiting Notch signaling has been shown to downregulate stemness-associated markers and encourage differentiation, thus reducing the malignancy of transformed cells (31).

In our investigation, we identified 16 CSC-related genes significantly associated with the EMT pathway in liver cancer. Numerous studies have confirmed that EMT facilitates liver cancer progression, reinforcing the validity of our identification of stem cell-related genes. Utilizing the expression profiles of these 16 identified genes, we applied the non-negative matrix factorization (NMF) algorithm to cluster analysis of LIHC samples from the TCGA-LIHC dataset. Notable differences in patient outcomes were observed across the groups, irrespective of whether the samples were categorized into two, three, or four clusters. To further investigate the underlying factors influencing outcome disparities, we conducted gene enrichment analysis, revealing significant enrichment of various well-established regulatory pathways related to tumor stemness in the samples of cluster 1, which included the VEGF and FGFR signaling pathways. This finding elucidates the reasons behind the poorer prognosis observed in this cluster.

The lack of clear diagnostic indicators often leads to late-stage diagnoses for many LIHC patients. To tackle this issue, our research focuses on developing diagnostic models for LIHC through various machine learning techniques. In the training dataset, our model demonstrated remarkable efficacy, achieving an AUC score of 0.999. To assess the effectiveness of our diagnostic approach, we analyzed two additional datasets, both of which consistently confirmed the robustness and reliability of our developed model. Compared to other machine learning algorithms, XGBoost not only demonstrates superior performance but also effectively manages the complexities inherent in biological data. Utilizing the XGBOOST algorithm, we identified S100A10 as a significant stem cell marker gene associated with the prognosis and progression of LIHC. Functional analysis further indicated that S100A10 is linked to cell proliferation and EMT in LIHC. Ultimately, we experimentally validated the expression and prognostic significance of S100A10 in LIHC.

In addition, our study was conducted through the analysis of multiple data sets, confirming the expression of the stem cell-related gene S100A10 in liver cancer, albeit based on a limited number of experiments. Consequently, our research has certain limitations. Future studies should aim to expand the sample size to better analyze the function of S100A10 in liver cancer and its relationship with stem cells. Additional experiments, including both cell-based and *in vivo* studies, are necessary to further elucidate the role of S100A10 in regulating stem cell mechanisms.

## 5 Conclusion

In summary, this study employed multi-omics analysis to investigate the role of stem cell-related genes in LIHC. These findings not only enhance our understanding of liver cancer biology but also offer new insights for developing personalized treatments and innovative immunotherapeutic strategies.

## Data Availability

The original contributions presented in the study are included in the article/supplementary material. Further inquiries can be directed to the corresponding author.
